# Model Selection Performance in Phylogenetic Comparative Methods Under Multivariate Ornstein–Uhlenbeck Models of Trait Evolution

**DOI:** 10.1093/sysbio/syac079

**Published:** 2022-12-28

**Authors:** Krzysztof Bartoszek, Jesualdo Fuentes-González, Venelin Mitov, Jason Pienaar, Marcin Piwczyński, Radosław Puchałka, Krzysztof Spalik, Kjetil Lysne Voje

**Affiliations:** Department of Computer and Information Science, Linköping University, Linköping, Östergötland, Sweden; Department of Biological Sciences, Florida International University, Miami, FL 33199, USA; IntiQuan GmbH, Basel, Switzerland; Department of Biological Sciences and the Institute of Environment, Florida International University, Miami, FL 33199, USA; Department of Ecology and Biogeography, Nicolaus Copernicus University in Toruń, Toruń, Kujawsko-Pomorskie, Poland; Department of Ecology and Biogeography, Nicolaus Copernicus University in Toruń, Toruń, Kujawsko-Pomorskie, Poland; Institute of Evolutionary Biology, Faculty of Biology, Biological and Chemical Research Centre, University of Warsaw, Warszawa, Poland; Natural History Museum, University of Oslo, Oslo, Norway

## Abstract

The advent of fast computational algorithms for phylogenetic comparative methods allows for considering multiple hypotheses concerning the co-adaptation of traits and also for studying if it is possible to distinguish between such models based on contemporary species measurements. Here we demonstrate how one can perform a study with multiple competing hypotheses using **mvSLOUCH** by analyzing two data sets, one concerning feeding styles and oral morphology in ungulates, and the other concerning fruit evolution in *Ferula* (Apiaceae). We also perform simulations to determine if it is possible to distinguish between various adaptive hypotheses. We find that Akaike’s information criterion corrected for small sample size has the ability to distinguish between most pairs of considered models. However, in some cases there seems to be bias towards Brownian motion or simpler Ornstein–Uhlenbeck models. We also find that measurement error and forcing the sign of the diagonal of the drift matrix for an Ornstein–Uhlenbeck process influences identifiability capabilities. It is a cliché that some models, despite being imperfect, are more useful than others. Nonetheless, having a much larger repertoire of models will surely lead to a better understanding of the natural world, as it will allow for dissecting in what ways they are wrong. [Adaptation; $AIC_{c}$; model selection; multivariate Ornstein–Uhlenbeck process; multivariate phylogenetic comparative methods; **mvSLOUCH**.]

The primary objective of phylogenetic comparative methods (PCMs) is to test evolutionary hypotheses whilst understanding and controlling for dependencies of trait measurements that arise due to shared ancestry and/or shared environmentally mediated selective pressures on macroevolutionary time scales. Evolution is a process that can include both deterministic (e.g., natural selection) and stochastic (e.g., numerous, small unmeasured selective forces and genetic drift) components. When the process includes speciation, the traits exhibited by extant species will all have spent some proportion of time evolving in common ancestral lineages.

For the past half-century, biologists have employed explicit stochastic processes to model trait evolution when phylogeny is involved. The generic goal is to use trait values measured at the tips of the phylogeny, along with estimates of the phylogenetic branching patterns to estimate parameters that capture the magnitude of the above-mentioned trait dependencies caused by these genetic and selective covariances. A Brownian motion (BM, Edwards 1970; Felsenstein 1985) was the first stochastic process proposed to model the evolution of continuously distributed traits on a phylogeny. The BM process’ variance, however, increases unboundedly with time and the process does not have a stationarity distribution. As such, a BM process is not well suited to model stabilizing selection around adaptive optima, a major mode of adaptive phenotypic evolution, where convergence of the variance and process to stationarity would be expected (Hansen and Orzack 2005). This requirement for mathematical models describing traits under selection was recognized by Felsenstein (1988); subsequently, Hansen (1997) demonstrated that by replacing the BM process with an appropriate Ornstein–Uhlenbeck (OU) process, the process’ variance converges. The Markovian, temporal homogeneity and mean-reverting properties of the OU process (under specific parameter classes) are well suited for modeling pervasive modes of natural selection such as stabilizing and directional selection. Furthermore, because it also satisfies Gaussian assumptions, parameter estimation through well-known techniques such as maximum likelihood is facilitated.

Following Hansen’s (1997) formulation, the initial focus was predominantly on univariate implementations of these methods, i.e., the study of single, well-defined traits, exemplified by, R (R Core Team 2019) packages such as **ape** (Paradis 2012), **geiger** (Harmon et al. 2008), or **ouch** (Ornstein–Uhlenbeck Models for Phylogenetic Comparative Hypotheses, Butler and King 2004). Biological traits, however, do not exist in isolation and both their form and function typically depend on interactions with other traits (Walsh and Blows 2009). The evolution of one trait cannot therefore be understood properly if one does not consider how other traits interact with the focal trait throughout its evolutionary history (e.g., Dobzhansky 1956; Chetverikov 1961; Frazzetta 1975; Lande and Arnold 1983; Wagner 1988). Multivariate extensions of the OU-based methods have been developed to analyze such trait interactions (e.g., Bartoszek et al. 2012; Beaulieu et al. 2012; Butler and King 2004; Clavel et al. 2015; FitzJohn 2010; Goolsby et al. 2017; Hansen et al. 2008; Ho and Ané 2014b; Mitov et al. 2019, to name a few). The early implementations, however, all run into issues with (1) long computational running time, (2) parameter identifiability due to large sample sizes required for accurate parameter estimation, and (3) computational complexity involved in obtaining the likelihood. These problems precluded large-scale simulation studies concerning model identifiability.

Fortunately, recent years have seen tremendous improvements in the computational capabilities of software for phylogenetic comparative methods. In particular, the **PCMBase** and **PCMBaseCpp** R packages (Mitov et al. 2020) provide a very efficient computational engine to obtain the likelihood for a wide class of phylogenetic Gaussian models. The Ornstein–Uhlenbeck family of models considered by **mvSLOUCH** (Bartoszek et al. 2012) is within the above class. Changing the likelihood calculation method resulted in massively reduced computational time for **mvSLOUCH**. This allows for considering larger sets of species; as our simulation studies will demonstrate, a phylogeny with 2000 tips does not pose any particular running time challenge. We also suspect that the dimensionality (i.e., number of considered variables) of each species can be much higher now. However, we have not run any tests in this direction, and the limiting factor will be the efficiency of eigenvector decomposition for matrices whose dimension equals the number of traits. One has to remember that increasing the number of traits induces a (quadratic in general) increase of parameters, hence would require larger samples. Understanding the number of estimable parameters as a function of observed species (and also tree height growth) is a topic for further work.

Importantly for the work here, the improvement in the algorithm has allowed us to design a large-scale simulation study to investigate the identifiability properties. In particular, we look at how well Akaike’s information criterion corrected for sample size ($AIC_{c}$, Hurvich and Tsai 1989) is able to distinguish between various multivariate phylogenetic Ornstein–Uhlenbeck models, corresponding to different adaptation and constraints hypotheses, for both simulated and empirical comparative datasets. We asked, for example, can causal claims of one trait driving another be defended against a null hypothesis of independent evolution?

To facilitate the reading, we have organized this work as follows. We begin with a brief justification of why we use the $AIC_{c}$ ( “On the use of AICc in model selection”) as our main model selection tool, then (“Model selection for the multivariate Ornstein–Uhlenbeck process in **mvSLOUCH**”) we introduce the multivariate Ornstein–Uhlenbeck process with a special focus on the possible forms of the drift matrix, A in Eq. (1). In this section, we also discuss model selection strategies and look into how well various OU setups can be differentiated by $AIC_{c}$, and how well **mvSLOUCH** recovers parameters of OU models. We then illustrate how in practise one can formulate and distinguish between competitive hypotheses concerning adaptive, evolutionary interactions between traits with two empirical studies (“Example analyses”). The first example concerns feeding styles and oral morphology in ungulates and the second fruit evolution in the genus *Ferula* (Apiaceae). We end the work with a discussion of the simulation findings in relation to the biological meaning inferred from the examples presented here. In the online Supplementary Material, we consider measurement error (Appendix SA), then we give more details on the possible parametrizations of the OU process’s drift matrix, Appendix SB, and in Appendix SC, and Appendix SD, we detail the setup and results of our simulation-­reestimation studies. Finally, in Appendices SE and SF, we report parameter estimates and other summary statistics from the ungulates and *Ferula* analyses. Included in **mvSLOUCH**’s vignette is another exemplary analysis on locomotion and forelimb morphology in carnivorans.

## On the Use of $AIC_{c}$ in Model Selection

It is important, especially for the applied reader, who wants to use these tools for making statements concerning empirical data, to know that information criteria (or in fact any statistical tool) can only reveal relationships between the models/hypotheses from a predefined set. If the correct model/hypothesis is not in this set (i.e., the user did not take it into consideration), then information criteria could point to models that are overparametrized (overfit). Examples of such situations could be errors in the phylogeny (topology or branch lengths), assumptions of homogeneity of the evolutionary process or incorrectly mapped shifts in parameters on the tree, and incorrect assumptions concerning structures of matrix parameters of the models. These issues stem from general principles of statistical inference and manifest in any study in any field that uses model selection. The only way that users may guard themselves against these issues is to carefully define the set of plausible hypotheses (according to subject matter knowledge) and to include both a simple “null” and a fully parameterized model in this set. Finally, information criteria rankings should not be treated as absolute truths. Rather they should be treated as guides to what level of information is contained in the data. Alternative models should also be looked at and their plausibility should be interpreted in light of the implied biological mechanisms. If information criteria point to the simplest, “null” model, then this could mean that there is too little information in the data for estimating parameters. On the other hand, the choice of the fully parametrized model could suggest that the “truest” model is not in the considered set of hypotheses. The estimated values under the fully parametrized model could be of some guidance. For example, if some estimated values are close to 0 or values for some levels of a parameter are very similar, then perhaps a model with, e.g. these values set to 0 or with these levels lumped together would be better supported by information criteria, and be more interpretable.

In our work here, we focus on the $AIC_{c}$ as it takes into account the number of observations. PCM studies often suffer from small samples, and also phylogenetically induced dependencies result in less independent data points than observed species (Bartoszek 2016), hence, giving some benefit of the doubt to more complex models could lead to consideration of more plausible hypotheses. One could also do model selection by, e.g., AIC or B(ayesian)IC; however, Bartoszek (2016) discussed that (unless the sample was very small) it is the likelihood that outweighed the “correction component” in the $AIC_{c}$, hence one should expect similar conclusions to be reached by alternative information criteria. However, we emphasize that there is no agreement in the literature on what is the most appropriate criterion for model selection and that bias toward more complex models has been observed in the literature, e.g., Ho and Ané (2014a) report that AIC and BIC tend to favor complex, with many shifts, scenarios of ancestral mappings of selective optima (levels of $\vec{\theta }$ in Eq. (1)). We do not consider reaching decisions based on $R^{2}$ ­values, nor likelihood ratio tests, the latter assume nested hypotheses, something that in general will not hold for the whole family of considered models.

## Model Selection for the Multivariate Ornstein–Uhlenbeck Process in mvSLOUCH

### The multivariate Ornstein–Uhlenbeck process

The multivariate Ornstein–Uhlenbeck process describes the evolution of a k-dimensional suite of traits $\vec{y}\in R^{k}$ over a period of time through the following stochastic differential equation


$$\begin{array}{l} d\vec{y}\left(t\right)=-A\left(\vec{y}\left(t\right)-\vec{\theta }\left(t\right)\right)dt+\Sigma_{yy}d\vec{W}\left(t\right), \end{array}$$ (1)

where $\vec{W}(t)$ is a k-dimensional standard Wiener process, $A\in R^{k\times k}$, $\vec{\theta }(t)\in R^{k}$, and $\Sigma_{yy}\in R^{k\times k}$. Setting A=0 reduces the model to a BM one. The OU process is a normal process, hence the joint distribution of all the traits on the phylogeny will be normal. From the biological perspective, the A matrix represents rates of adaption (if all its eigenvalues have positive real part) of trait values towards optimum values, $\vec{\theta }\left(t\right)$ the primary optimum (sensu Hansen 1997) trait values at time t, whereas $\vec{W}\left(t\right)$ and $\Sigma_{yy}$ represent the non-directed stochastic perturbations caused by unconsidered selective factors, environmental fluctuations or constraints due to, for example, developmental correlations. In addition, $\vec{\theta }\left(t\right)$ can take the form of a step function representing fixed selective regimes painted on phylogeny. The A matrix generalizes the “α parameter” of a one-dimensional OU process (e.g., Hansen 1997; Butler and King 2004) to the multivariate case. In the single-trait case, only the rate of adaptation was present but, in the multi-trait case, we need to include the interactions between the traits on their path to their primary optimum, and these interactions are described in A’s eigenvectors.

The possibility to work with a wide class of A matrices and to be able to impose various restrictions is highly relevant for evolutionary studies based on the OU process. Different setups (e.g., diagonal, symmetric positive definite, upper, lower triangular) will have different biological interpretations concerning causation (i.e, selective covariance patterns, see Bartoszek et al. 2012; Reitan et al. 2012). To correctly interpret the various parameterizations of A and $\Sigma_{yy}$, one important distinction must be made: evolution and adaptation cannot be treated synonymously. The observed selection can be either direct, when it is causally related to the trait under study, or indirect, when it acts through other correlated traits. The trait, indirectly affected by selection, evolves, but not adaptively. Taking into account this remark, the general interpretational framework for OU models (with all eigenvalues of A having positive real part) here can be as follows:

(a) diagonal A and diagonal $\Sigma_{yy}$—traits evolve independently, for example as belonging to different developmental modules,
(b) diagonal A and non-diagonal $\Sigma_{yy}$—traits are related through the $\Sigma_{yy}$ matrix indicating correlated evolution due to, for example, developmental constraints or covariation with other unmeasured traits under selection,
(c) non-diagonal A and diagonal $\Sigma_{yy}$—traits evolve adaptively and they may serve as primary optima for each other; here, for example, a symmetric A may indicate trait-trait adaptive co-evolution.
(d) non-diagonal A and non–diagonal $\Sigma_{yy}$—in this scenario, there is a combination of non-adaptive and adaptive trait evolution; here, the interpretation depends strongly on the tested hypothesis, see “Example analysis 2”.

Singular, det(A)=0, matrices will have a very specific meaning—some linear combination of traits evolves as a Brownian motion, and there is no tendency to move towards an optimum along some directions in the trait space. Ancestral effects, in this case, do not disappear with time, leading to non-adaptive evolution (in some directions). After the original Brownian motion process, the model implemented in the **slouch** package (Hansen et al. 2008) and then generalized to the multivariate setting by Bartoszek et al. (2012) are the first allowing for such a singularity,


$$\begin{array}{l} \begin{array}{ll} & d\vec{y}(t)=-A({\vec{y}}_{t}-(\vec{\psi }(t)+Q\vec{x}(t)))dt+\Sigma_{yy}d{\vec{W}}_{y}(t)\\ & d\vec{x}(t)=\Sigma_{xx}d{\vec{W}}_{x}(t), \end{array} \end{array}$$ (2)

${\vec{W}}_{y}(t)$ and ${\vec{W}}_{x}(t)$ are independent multivariate Wiener processes. We can see that, in the language of Eq. (1), we will have


$$\begin{array}{ll} A_{Eq.(1)}= & \left[\begin{array}{ll} A_{Eq.(2)} & \;-A_{Eq.(2)}Q\\ 0 & \;0 \end{array}\right],\;\vec{\theta }\left(t\right)=\left[\begin{array}{l} \vec{\psi }\left(t\right)\\ \;\vec{0} \end{array}\right],\\ & \;\Sigma_{yy}^{Eq.(1)}=\left[\begin{array}{ll} \Sigma_{yy}^{Eq.(2)} & \;0\\ 0 & \Sigma_{xx}^{Eq.(2)} \end{array}\right]. \end{array}$$

### Model Selection Strategies

Given the plethora of possible models to consider in **mvSLOUCH**, a methodological approach is necessary to choose the model best supported by the data. The statistically sound approach is to first design a collection of models informed by biologically plausible hypotheses. They are expressed in the **mvSLOUCH** framework as appropriate parametrizations of the A and $\Sigma_{yy}$ matrices, furthermore enhanced by the possibility to fix specific entries of these and other parameters to a user provided value. For each provided setup, maximum likelihood estimation by **mvSLOUCH**, through the underlying **PCMBase** computational engine, is performed. Then, the best supported model according to the desired criterion is chosen.

The simplest pair of hypotheses to test statistically is whether the traits evolve as a Brownian motion $\left(A_{Eq.(1)}=0\right)$ or not $\left(A_{Eq.(1)}\;\neq \;0\right)$. This tests whether the traits are randomly diffusing through time (due to both neutral processes as well as numerous, small unmeasured selective forces acting in different directions) or adapting (if $A_{Eq.(1)}$ ’s eigenvalues all have positive real part) to some deterministic optimum. However, there are more complex setups to test, if we look at Eq. (1) then, as already discussed in the bullet points following the equation, imposing different structures on A and $\Sigma_{yy}$ will lead to different adaptive hypotheses, described in Appendix SB. One has to remember that a wrong model can be chosen due to the effect sizes of parameters. For example, sometimes a BM model could be favored over a true OU process, if the latter has close to 0 values in $A_{Eq.(1)}$. Looking at the half-lives ($t_{i_{0.5}}=ln\;2/\lambda_{i}\;$, where $\lambda_{i}$ is an eigenvalue of A, see also Bartoszek et al. (2012), of an OU process will tell us if we should expect adaptive effects over our observed time-scale, tree height).

Once we have the values of the information criteria for a collection of models representing our hypotheses, comes the question whether we should only consider the best supported model or look also into (some of) the alternative ones. Burnham et al. (2011) write that plausible models are included within a difference in $AIC_{c}$ values up to 7 and implausible models will have an $AIC_{c}$ value greater than about 14. When the difference in $AIC_{c}$ values is between 7 and 14, then “evidence … is inconclusive and value judgments for hypotheses in this region are equivocal” (caption of Fig. 2 in Burnham et al. (2011)). It has to be pointed out that Burnham et al. (2011) did not consider phylogenetically structured data, hence their conclusions should be treated as suggestions. The effects of a hierarchical correlation between data points on the $AIC_{c}$ still needs to be explored and in effect rules of thumb still need to be developed for PCMs (or more generally hierarchically correlated data). We advocate that a user strikes a balance between what the formal statistics says and what biological intuition says. Instead of giving a single definitive answer, the user should discuss the different possibilities and their implications.

Therefore, there is an incentive to explore the implications of less supported models, but with relative $AIC_{c}$ values not much larger than the best one. It is a question of what is meant by “much larger” for a multivariate PCM setting, as a definite study on this matter is yet to be presented. Hence, rules of thumb intuitions, based on a single response and independent data, have to be cautiously generalized. Most importantly, when deciding on a model’s plausibility, subject matter knowledge needs to be taken into consideration. There need to be other arguments supporting the consideration of the best model and/or alternatives. In particular, this should be strong domain-specific knowledge that indicates underlying mechanisms and understanding of the models. One has to very carefully balance what the data is saying and what we know/suspect prior to the study; automatized decisions either way are not the optimal approach. One should look at the estimated parameter values themselves, their “effect size” and if they are of sensible magnitudes. Extreme values, e.g., nearly 0, could indicate that alternative models, where the particular parameter is, e.g., not present, could be better supported by the data. Having decided on a set of plausible models, one can perform a simulation study, as we did in our “Example analysis 2” to see if it is possible to distinguish between them. Afterward, the conclusions about the system should be presented in the light of the accepted models, what they agree and disagree on, and what models are distinguishable. We cannot forget that all conclusions are relative to the set of models considered by us (each one corresponding to some hypothesis). It could very well be that a model (hypothesis) outside the considered set would fit (be supported) better to (by) the data, i.e., models can be wrong but some will be more wrong than others. In summary “hard thinking” (p. 29 Burnham et al. 2011) is required for comparing between models so that “analysts make an informed choice from all available strategies, employing each in contexts where they are most informative.” (Steidl 2006).

### Model Selection Efficiency and Quality of Parameter Estimates

Here we perform an extensive simulation study to investigate how well the new **mvSLOUCH** package is able to perform model selection. The tree is simulated as a pure birth tree using *TreeSim::sim.bd.taxa()* (Stadler 2009, 2011) for a number of tips, n=32, 64, 128, 256, 512, 1024, 2048. For comparison between all the simulations, all the phylogenies are rescaled to height 1. Four dimensional trait data is simulated under BM, OUOU (no traits evolve marginally as BMs, A in Eq. (1) is non-singular) and OUBM (some traits evolve marginally as BMs, Eq. 2) models and are then recovered under various assumptions. $AIC_{c}$ was used to distinguish among the various models. The simulation-reestimation setups considered are presented in Appendix SC. Under the OUBM models, the estimation procedure is such that the compound, in terms of Eq. (2)’s formulation parameter, $B=-A_{Eq.(2)}Q$ is estimated directly in the numerical optimization of the likelihood. In the old, slow version of **mvSLOUCH,** this parameter was estimated via an iterated GLS, but after experimenting we found that this approach’s performance was much poorer both in terms of model selection ability and parameter recovery.

In Table S.2 in Appendix SC, we present how **mvSLOUCH** is able to select the correct model. For small trees (32, 64 tips), there is some bias of the OUOUs2 model (non-diagonal A, diagonal $\Sigma_{yy}$, see Table S.1 for definition of model abbreviations) towards simpler models, like BM or OUBM. However, we also noted another type of bias. For all tree sizes, the simpler OUOUs1 model (diagonal A, diagonal $\Sigma_{yy}$) is always preferred over the more complicated OUOUs2 model. The OUBM models perform much better with respect to distinguishing between competing hypotheses. For small trees, the simpler OUBMs1 (diagonal A, diagonal $\Sigma_{yy}$) model is preferred over the more complex OUBMs2 (non-diagonal A, diagonal $\Sigma_{yy}$) one. As the tree size grows, this preference diminishes. Model selection under the OUBM model is not perfect, but one can observe that the tendency is toward the correct model. On a coarser classification, BM versus OUOU versus OUBM, no problems are noticed. Even for the smallest setup, 32 tips, models are correctly classified. Sometimes a BM can be misclassified as an OUBM but the chances of this decrease with the number of tips.

Following the generally optimistic results concerning **mvSLOUCH**’s model selection abilities, we take a more detailed look into how well it can recover model parameters and certain important compound parameters. Recovery of parameters of a phylogenetic OUOU process is known to be a difficult problem. Cressler et al. (2015) and Ho and Ané (2014a) considered the one dimensional phylogenetic OU process, whereas Pienaar et al. (2008) and Bartoszek et al. (2012) analyzed the phylogenetic OUBM process. To evaluate the quality of parameter estimates, the following error formulæ were introduced


$$\begin{array}{l} Euclidean\;estimation\;error=\sqrt{{\left(\vec{x}-\hat{\vec{x}}\right)}^{T}(\vec{x}-\hat{\vec{x}})}, \end{array}$$ (3)

and


$$\begin{array}{l} Relative\;estimation\;error=\frac{\sqrt{{\left(\vec{x}-\hat{\vec{x}}\right)}^{T}\left(\vec{x}-\hat{\vec{x}}\right)}}{\sqrt{{\vec{x}}^{T}\vec{x}}}, \end{array}$$ (4)

where $\vec{x}$ and $\hat{\vec{x}}$ are, respectively, the vectorized version of the true value of the given parameter and its estimate. The Euclidean distance is for the case when the parameters to be estimated equal $\vec{0}$, these are the ancestral states and deterministic optimum values. For matrix or vector parameters, we do not compare entry by entry, but jointly over all positions. Through such an approach, we obtain the possibility to look at the matrix/vector parameter as a whole and not entry by entry, which could miss potential interactions between estimates. This could be especially relevant when the given matrix is parametrized through a decomposition (e.g., when it is assumed to be eigendecomposable with real positive eigenvalues). The optimization is over the eigenvalues and eigenvectors and hence one cannot expect to have independence between the estimates of the actual matrix entries. In Figs. S.1–S.9, we present boxplots for different values of the number of tips, n, for the most relevant parameters for the given models. These include the ancestral state (all models), the deterministic part of the primary optimum (OUOU, OUBM), $\Sigma_{yy}$ (all models), $\Sigma_{xx}$ (OUBM), Σ (all models), A with eigenvalues and half-lives (OUOU, OUBM), Q (OUBM), evolutionary and optimal regressions (OUBM), trait regressions and limiting (long-term) trait regressions (OUOU), and between traits correlation and stationary correlation matrices (OUOUs2 and OUBM).

We can see that for all setups and parameters with n the error either decreases or tends to stabilize. No systematic increase of error with n is observed. For the BM model, the diffusion matrix seems well estimable, whereas the ancestral state’s error does not decrease (in line with the observation that it cannot be consistently estimated, Ané 2008; Sagitov and Bartoszek 2012). For the OUOU model, we can see that estimation is much better in the OUOUs1 case, i.e., the traits are independent. Error in A’s estimation decreases with sample size. When moving to the OUOUs2 case—traits dependent through a non-diagonal A—there are many more outliers with large estimation error in A (and also its eigenvalues and half-lives). The diffusion parameters $\left(\Sigma_{yy},\Sigma \right)$ and deterministic optimum $\left(\vec{\psi }\right)$ are much better estimated. Parameters that depend on both the drift and diffusion (regressions and correlations) exhibit the same sort of outliers that the estimator of A does. In the OUBM models’ case, we can see similar behavior, diffusion parameters are well estimated, the drift ones are more difficult. However, Q seems to be well estimated—error surely decreasing with n.

However, we can also observe another trend. As the number of tips of the tree increases, errors in estimation procedures become more common (Table S.4), especially for the Brownian motion model which has closed form formulæ. We take a deeper look at this in our “Example analysis 2,” where this becomes more dramatic and clear.

## Example Analyses

### Example Analysis 1: Feeding Styles and Oral Morphology in Ungulates

Hypsodonty is a classical feature linked to the study of feeding adaptations in mammals (e.g., Van Valen 1960; MacFadden 1992; Strömberg 2006). It essentially corresponds to a measure of relative crown height that reflects the capabilities of the tooth to wear longer for processing abrasive material such as grasses with high silicophytolith contents, or soil and grit that can be ingested during feeding (Mendoza et al. 2002; Damuth and Janis 2011; Jardine et al. 2012). Here we show the advantages of studying this classical feature under the capabilities offered by **mvSLOUCH**.

The first, most obvious, advantage is the possibility of testing the adaptive significance of hypsodonty in a comparative framework consistent with the process of natural selection. When introducing the model of adaptive evolution that we extend here, Hansen (1997) showed that the comparative study of hypsodonty should not be limited to a statistical correction due to phylogeny (Pérez–Barbería and Gordon 2001) but rather used to account for the selective history of species to estimate current adaptation. In this seminal OU formulation, Hansen (1997) specified a selective regime by mapping the emergence of a new primary optimum in the equid tree, distinguishing browsing and grazing niches. Further extensions allowed for not only specifying more complex regimes but also for comparing their adaptive significance through information criteria (Butler and King 2004), as currently implemented by **mvSLOUCH**. This setup allows us to compare browsers and grazers with another niche: the mixed feeders, i.e., those ungulates that both browse and graze (Mendoza et al. 2006; Mendoza and Palmqvist 2008; Damuth and Janis 2011). The character assignation of these three feeding styles was based on Pérez–Barbería and Gordon (2001) for the present example.

**mvSLOUCH** also offers the possibility of studying hypsodonty without the need for computing ratios. To study trends in molar crown height free of the effects of general body size, hypsodonty has often been studied as a ratio (the hypsodonty index), typically dividing the crown height by some other linear variable (Van Valen 1960; Janis 1988; Mendoza and Palmqvist 2008). However, ratios have been criticized for their poor distributional properties and limited usefulness as size-adjusted variables (Reist 1985; Albrecht et al. 1993; Mendoza et al. 2002), whereas regression techniques have been recommended for scaling purposes (Packard and Boardman 1999; Garcìa-Berthou 2001; Freckleton 2002). In the context of this example, a regression setup implies using crown height (a numerator of the hypsodonty index) as a response variable with molar width (a denominator of the hypsodonty index) as a continuous covariate (an explanatory variable). The specification of a continuous predictor under the adaptation-inertia framework is not as simple as under a regular regression, however, because it requires information on both the present trait values (at the tips) as well as their past history (across the tree). The first way of dealing with this problem was to assign fixed covariate values directly on tree branches (Hansen 1997). A more elaborate and flexible solution, as explained above, models the covariate as a Brownian motion (OUBM model) or Ornstein–Uhlenbeck (OUOU model) process, without fixing trait values on tree branches (Hansen et al. 2008; Bartoszek et al. 2012). These models (OUBM and OUOU) can be contrasted under the new version of **mvSLOUCH** with an alternative in which the traits do not evolve toward an optimum (BM). This comparison was conducted for ungulates using the unworn lower third molar crown height ($HM_{3}$, measured from the base of the crown to the tip of the protoconid) and width ($WM_{3}$, measured at the occlusal surface, between the outer aspects of the protoconid and the entoconid) data presented by Mendoza et al. (2002). We used logarithmically transformed variables (originally measured in cm) for data analysis (Pérez–Barbería and Gordon 2001).

A final advantage, which lies at the core of **mvSLOUCH**, is its multivariate nature. This is especially true for a structure such as the skull, which can be subject to multiple, often conflicting, selective pressures (Porto et al. 2009; Damuth and Janis 2011; Toljagić et al. 2018). The ungulate feeding apparatus in particular can be seen as reflecting the requirements for either processing or selectivity of food (Gordon and Illius 1988; Janis and Ehrhardt 1988; Pérez–Barbería and Gordon 2001). Hypsodonty optimizes the former by increasing chewing effectiveness, ensuring that the animal can process food fast enough to meet its energy demands (Pérez–Barbería and Gordon 1988a). A narrow focus on hypsodonty can, however, clarify the role of molar teeth in food comminution whereas the implications of changes in oral morphology for food selectivity remain unexplored (Gordon and Illius 1988). We explore these implications here by specifying a bivariate model in which a variable informative of food selection is used as a response along with crown height. We use muzzle width (MZW, measured at the outer junction of the boundary between the maxilla and premaxilla) where narrower muzzles facilitate greater selectivity in food foraging (Janis and Ehrhardt 1988; Mendoza and Palmqvist 2008). As with the tooth variables, muzzle width (originally measured in cm) was obtained from Mendoza et al. (2002), and was logarithmically transformed for data analysis.

We used the mammalian tree presented by Hedges et al. (2015) after pruning it to only include the 104 ungulate species for which we had phenotypic data available (i.e., for which we had complete information of all the variables at the species level). This tree resulted from a synthesis of studies in molecular evolution and phylogenetics that incorporated a time calibration component (Hedges et al. 2006; Hedges and Kumar 2009). Diet was assigned as an adaptive regime by ancestral state reconstruction under stochastic character mapping (Nielsen 2002; Nielsen and Bollback 2003; Bollback 2006), as implemented in the **phytools** R package (Revell 2012), after identifying the best supported transition rate model (equal rates, symmetric backward and forward rates, all-rates-different). As explained above, the full regime specification ($OU_{F}$) included three diets (browsers, grazers, and mixed feeders). Considering the possibility that the craniodental variables may respond to simpler selective regimes, we specified two binary alternatives by lumping mixed feeders with either browsers ($OU_{G}$) or grazers ($OU_{B}$). We also ran analyses under a single regime for the entire tree for comparison ($OU_{1}$), which would be an indication that diet has little adaptive significance for the morphological traits under consideration. The compiled dataset (phenotypic data and phylogenetic tree) and analysis scripts can be found in the accompanying GitHub repository.

For each of these regime specifications, we conducted a comprehensive model comparison under the new wrapper function of **mvSLOUCH**, which runs a series of multivariate models (BM, OUBM, OUOU) under different specifications of the underlying parameters. For the OUOU and OUBM models, we ran each setup five times from different starting points (however, we would encourage users to try more starting points as we do in “Example analysis 2”). The model setups consisted of all possible combinations of predefined A (diagonal, upper or lower triangular, and decomposable with positive or real eigenvalues) and $\Sigma_{yy}$ (diagonal and upper triangular) classes (with the diagonal of A always specified to be positive). Model support was assessed through $AIC_{c}$ with a difference larger than two units providing support to the candidate with lower value (Burnham and Anderson 2004).

**Figure 1.**
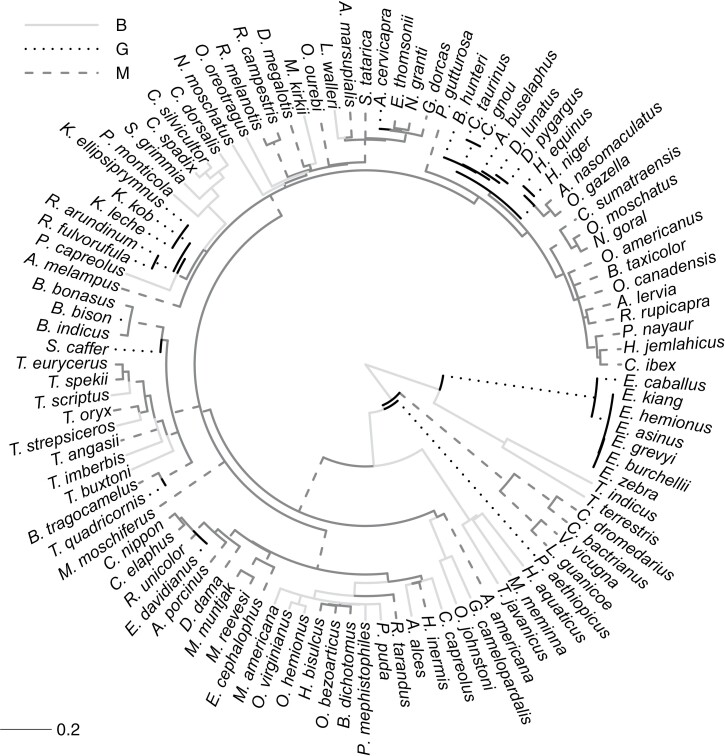
Ungulate phylogeny with feeding preferences (B = browser; G = grazer; and M = mixed feeder) reconstructed under 500 stochastic character mappings with a symmetric transition matrix (Appendix SE1). Character state transitions were assigned to those branches where the highest posterior probability shifted from one diet category to another. Branch lengths are scaled and thus informative of relative time (scale provided at the bottom left). Full species names are provided in a higher resolution color phylogeny in Fig. S.19 in Appendix SE1.

**Figure 2.**
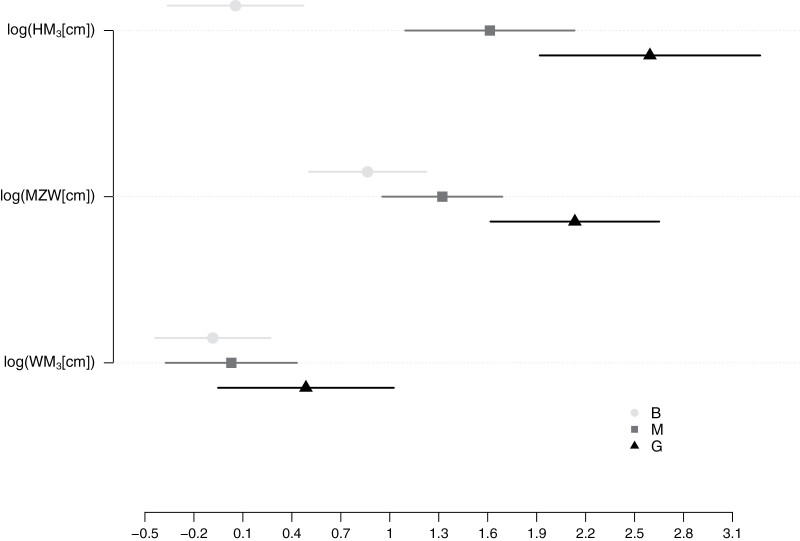
Optima (with 95% CIs estimated by GLS conditional on A and diffusion matrix parameters) for lower third molar crown height ($HM_{3}$), muzzle width (MZW), and lower third molar crown width ($WM_{3}$) under the best supported model (after logarithmic transformation). This model corresponds to an OUOU with three feeding strategies specified as selective regime (B = browser; M = mixed feeder; G = grazer). Note that the only feeding style showing no overlap in CI is browsing under $HM_{3}$. Nevertheless, the optimum estimates of both grazers and mixed feeders are excluded from each other’s CI except for $WM_{3}$ (where no optimum estimate is clearly differentiated), indicating that they are differentiable, albeit not as distinctively as browsers. For numerical outputs, as well as estimates under other models, see Appendix SE3.

We found that for all regime specifications ($OU_{F}$, $OU_{G}$, $OU_{B}$, and $OU_{1}$), including those based on the evolutionary history of diet (Fig. 1), OUOU was preferred with a model setup consisting of diagonal A and upper triangular $\Sigma_{yy}$. Given that diagonal A OUOU models deserve some caution (see “Model selection efficiency and quality of parameter estimates”), we also explored the best alternative candidates for comparison. The best alternatives, for all the regime specifications, were OUOU models with upper triangular $\Sigma_{yy}$ and non-diagonal A. However, both lower and upper triangular A matrices were equally supported in all cases, providing no clear preference for any alternative model (see Table S.9 in Appendix SE2). This lack of consistency, combined with the lower support for these alternative models (all of them were more than four $AIC_{c}$ units higher than the preferred models), suggest that the off-diagonals of A are not highlighting any relevant trend in the data. As a result, the diagonal A parametrization, rather than a statistical artifact linked to bias, can be considered appropriate for this dataset. There is substantial support for models describing feeding adaptations, particularly the full regime specification (Table 1). This result holds true for both the best (diagonal A) and alternative (non-diagonal A) candidates of the model set (see Table S.9 in Appendix SE2), providing stronger support for the full regime hypothesis. The preferred model highlighted a positive association between the morphological variables (Table 2), with browsers showing the lowest primary optima in all cases, and grazers the highest (Fig. 2). The optima can be more clearly differentiated for crown height and muzzle width, especially the former, with browsers showing distinguishably lower primary optima. This explains why the regime that keeps this feeding style in a separate niche ($OU_{B}$) is much closer in support to the preferred model ($OU_{F}$, $\Delta AIC_{c}=3.66$) than the alternative lumped regime ($OU_{G}$, $\Delta AIC_{c}=23.62$). It needs to be pointed out that for reproducibility the analyses were run with a specific random seed set.

**Table 1:** Comparison of the best candidates under each model type showing statistics of model support ($AIC_{c}$ and $R^{2}$) and phylogenetic half-lives (reported as percentage of tree height in the eigenvector directions, ${\vec{e}}_{i}$). The full regime specification ($OU_{F}$) is the best supported model with lowest $AIC_{c}$ and highest $R^{2}$. Other parameter estimates for these models are shown in Appendix SE3.

			Half-lives (%)		
Model	$AIC_{c}$	$R^{2}$	${\vec{e}}_{1}$	${\vec{e}}_{2}$	${\vec{e}}_{3}$
BM	96.65	0.07	–	–	–
$OU_{1}$	91.59	0.01	34.25	36.05	189.11
$OU_{F}$	57.52	0.19	23.28	31.13	38.92
$OU_{G}$	81.14	0.07	26.76	33.57	69.25
$OU_{B}$	61.18	0.15	29.15	35.26	47.70

**Table 2:** Observed correlations between traits and evolutionary regression coefficients with 95% parametric bootstrap (1000 bootstrap replicates) confidence intervals (CI, within parenthesis), which in all cases are significantly positive (intervals exclude zero). However, the correlation between the responses ($HM_{3}$ and MZW) conditional on the covariate ($WM_{3}$) is weaker (0.24) and non-significant (CI −0.04,0.655).

Trait	Correlations		Regression
	MZW	$WM_{3}$	$WM_{3}$
$HM_{3}$			
	0.64 (0.009,0.749)	0.65 (0.003,0.748)	0.73 (0.003,1.003)
MZW	——	0.81 (0.007, 0.871)	0.9 (0.007, 1.374)

All things considered, the preference for a full regime specification ($OU_{F}$) for the ungulate dataset reflects the adaptive significance of feeding style on oral morphology. The only alternative plausible hypothesis ($\Delta AIC_{c}<4$) is the regime distinguishing browsers from the rest ($OU_{B}$), which still reflects the importance of diet as a selective factor. Importantly, a closer inspection of the optima shows that browsers exhibit a consistent trend across models (Table S.13) and that, despite less differentiated, grazers and mixed feeders constitute distinctive niches (Fig. 2). From this comprehensive point of view, the plausible support for $OU_{B}$ (Burnham et al. 2011) does not preclude the relevance of mixed feeders and grazers as separate niches, but highlights the resemblance of their oral morphologies relative to browsers. The strong support for the OUOU model highlights the role of stabilizing selection on each of the variables considered. Despite the large influence of selection on the pattern, adaptation is not instantaneous, with all half-lives surpassing 20% of total tree height. This confirms slow evolution that might result from morphological constraints acting on ungulate craniodental attributes (Janis and Ehrhardt 1988; Damuth and Janis 2011; Toljagić et al. 2018).

Given that the data set is influenced by the effects of both adaptation and inertia, neither approaches ignoring phylogeny nor those that simply assume that phylogenetic inertia is present (such as BM) are appropriate for analyzing it (Table 1). For example, Pérez–Barbería and Gordon (2001) analyzed differences in body size and oral morphology between ungulates with different feeding styles, using comparative methods focused on controlling for phylogenetic effects. Muzzle width (as well as other oral traits) showed differences when phylogeny was ignored, whereas only hypsodonty attributes and body mass showed differences when comparative methods were used. It is not surprising that the feeding associations hinted by the non-phylogenetic analyses were deemed non-existent by the comparative methods, given that approaches focused on controlling for phylogeny can remove the adaptive signal of patterns in which the traits and the environment share some history (Hansen 2014). Hypsodonty might have been an exception due to a strong association with feeding styles, which makes sense considering how well crown height differentiates feeding optima (Fig. 2). Nevertheless, they concluded that feeding adaptations for most oral traits (including muzzle width) were subsumed by the effects of body mass and shared ancestry.

Our results suggest, however, that scaling effects and shared ancestry in ungulates are not inconsistent with feeding adaptations in oral morphology. All traits covary positively (Table 2) with optima that exhibit a similar trend (Fig. 2) possibly reflecting scaling effects. The conditional correlation of crown height and muzzle width is weaker than the general correlations (Table 2) suggesting that, as expected for dental measures, the explanatory variable can work effectively as a scaling factor (Gordon and Illius 1988; Shipley et al. 1994; Damuth and Janis 2011). Under the preferred OUOU model, an adaptive pattern dominated by the scaling factor would be corroborated through a non-diagonal A (in particular, an upper triangular matrix with variables ordered as in Appendix SE3, Table S.11). And yet, the preferred model has a diagonal A matrix, suggesting that all traits are adapting independently to feeding preferences. This result is more consistent with the idea of allometric patterns arising from natural selection on more than one trait (Lande 1979), than with the idea of feeding adaptations subsumed by the effects of body size (Pérez–Barbería and Gordon 2001).

In our results, where general phylogenetic effects are not removed from the adaptive pattern, both crown height and muzzle width are important components of feeding preferences in ungulates (Janis 1988; Janis and Ehrhardt 1988; Mendoza and Palmqvist 2008). Feeding differences obtained from crown height alone would downplay the interspecific relevance of food selection (linked to muzzle width) compared with food processing (Pérez–Barbería and Gordon 2001). Also, because hypsodonty is not strictly associated with obligate grazing, focusing on relative crown height alone suggests that the evolution of oral morphology in ungulates involves adaptations for broadening the feeding niche (Feranec 2003). That both traits adapt to primary optima, however, is more indicative of a functional trade-off reflected by a consistent opposing trend in the distribution of oral trait optima across feeding types (Fig. 2). Hypsodonty is particularly advantageous when exhibiting a broad multiridged surface that facilitates processing flat blades of grass during mastication (Pérez–Barbería and Gordon 1988a, 1988b). But a relatively flat occlusal surface is not advantageous for cutting material of different physical properties (e.g., dicotyledonous leaves or twigs), and then suboptimal for browsing (Damuth and Janis 2011). Similarly, a broad muzzle is advantageous for grazers by enabling them to take large bites of food that is usually of low nutritional value (i.e., grass), and therefore requires a high intake rate for meeting energy demands (Janis 1988; Janis and Ehrhardt 1988; Feranec 2003). It is not so advantageous, however, for animals such as browsers that benefit from picking out certain plants or plant parts, as well as reaching areas concealed by structures such as thorns and spines (Gordon and Illius 1988; Janis and Ehrhardt 1988; Mendoza and Palmqvist 2008). Therefore, the attributes exhibited by browsers that optimize food selection (small size, low-crowned teeth, narrow muzzles) can be seen as traits maintained by stabilizing selection (Hansen 1997) that are no less adaptive than the attributes that optimize food processing (Feranec 2003). The latter (i.e., attributes optimizing food processing), exhibited by grazers, follow exactly the opposite trend (large size, high-crowned teeth, broad muzzles) reflecting conflicting functional demands that result in well-adapted forms for each feeding type (Gordon and Illius 1988; Damuth and Janis 2011). The mixed feeders show intermediate optima for all traits making them, more than transitional grazers, forms that optimize both functions (Fig. 2: CI overlap in crown height with grazers and in muzzle width with browsers, even when browsers and grazers themselves show no overlap in either trait) without specializing for either. Given that mixed feeders experience resource fluctuations over the seasons (Gordon and Illius 1988; Mendoza et al. 2006; Damuth and Janis 2011), specialization in either way (processing or selectivity) could be disadvantageous for this feeding style. As explained earlier, however, our results show a stronger resemblance between mixed feeders and grazers, suggesting that the oral morphology of the former is better adapted to intake rate than to food selection.

### Example Analysis 2: Fruit Evolution in *Ferula* (Apiaceae)

Seed size is a trait that has been known to affect multiple aspects of plant ecological strategies (Moles 2018). Selection for a particular seed size often requires the coordinated evolution of seed or fruit traits facilitating a particular ecological strategy. For example, the intensity of seed predation may increase with changes in seed size driving selection for higher investment in seed or fruit defensive traits (Fricke and Wright 2016). Similarly, there is a strong allometric relationship between seed/fruit size, area, shape as well as mass of dispersal appendages such as wings, pappus or kapok that directly influence terminal velocity (Niklas 1994; Greene and Quesada 2005). In extreme cases, when seed size exceeds a certain threshold, the mode of seed dispersal may change, triggering the evolution of novel seed dispersal appendages. Anemochory (wind dispersal), for instance, is almost invariably limited to cases where the seed mass is less than approximately 1 g, whereas zoochory (animal dispersal) characterizes large-seeded species (Tackenberg et al. 2003; Greene and Quesada 2005). These observations have led many authors to conclude that the structural properties of fruit and seed are subject to strong selective pressures and have been shaped by a trade-off among three primary functions: seed provision, seed protection, and seed dispersal. However, the evolutionary consequences of this statement concerning changes in fruit and seed structure have not been tested in a phylogenetic framework.

To test the evolutionary consequences of potentially conflicting selection pressures on three primary functions (dispersal, protection, and provision) of seeds, we examine fruit variation in the umbellifer species-rich genus *Ferula* (including *Dorema* and *Leutea*, Kurzyna-Młynik et al. 2008). In Apiaceae, the fruits are dry schizocarps and the dispersal units are single-seeded mericarps. The mericarp is usually tightly connated with the seed taking over the protective role of the seed coat and determining the mode of dispersal. The fruit anatomy in the family is structured according to a common plan (Fig. S.20), although a wide array of modifications can be encountered (Wojewódzka et al. 2019). In the genus *Ferula*, fruits are dorsally compressed with marginal ribs developed into wings suggesting wind as a main dispersal agent (Fig. S.20). The degree of wing development varies among species indicating potential correlation with fruit size (Fig. S.20). Protective characters, such as the periderm (fruit wall) thickness and the number and arrangement of oil ducts (vallecular vittae), also change considerably among species making the genus *Ferula* an interesting model system for studying fruit evolution. For the purpose of this study, we measured five characters on fruits for *Ferula* species. The periderm thickness and the proportion of oil ducts covering the space between median and lateral ribs were assigned to protective functions, wing thickness, and wing area to dispersal functions, whereas fruit mass was used as a proxy of seed provision (Fig. S.20).

The maximum likelihood phylogenetic tree (deposited in the Supplementary Material) with 78 tips was obtained from the recent molecular taxonomic revision of the genus (Panahi et al. 2018) and dated using a secondary calibration point for the root based on Banasiak et al.’s (2013) work with **mcmctree** (Yang 2007). Such a small sample size is, to say the least, problematic considering the dimensionality of the models we want to consider. However, our main aim here is to show the modeling possibilities that **mvSLOUCH** has, i.e., that one can declare a family of models that corresponds to informed biological hypotheses. When analyzing the results, we will be able to observe what issues can arise given the sample size. For ease of reproduction, the analyses were run with very specific random seeds.

**Figure 3.**
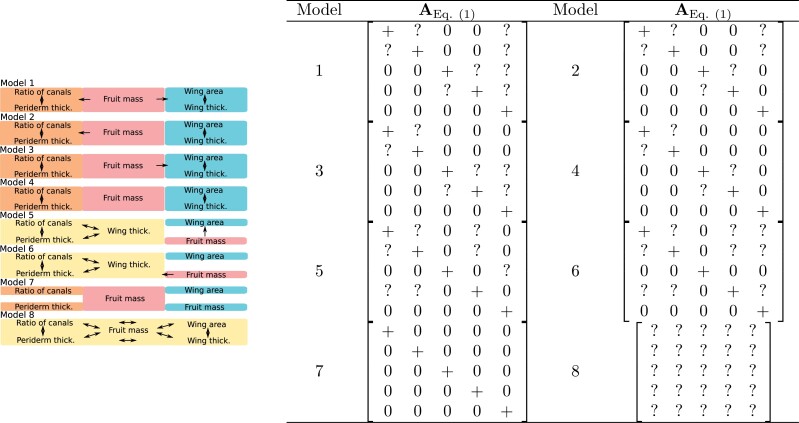
*Left*: The graphical representation of tested models. “The functional allometry model” is represented by model 1, “parcellation models” by models 2, 3, and 4, whereas the developmental allometry models by models 5 and 6. In models 1, 2, 3, and 4, the functional modules (dispersal: wing thickness and wing area, protection: periderm thickness and ratio of canals, and provision: fruit mass) are colored. Traits within protective and dispersal modules affect each others optima (double-headed arrows). Fruit mass, on the other hand, affects traits belonging to other modules (single-headed arrows). In the developmental allometry models, periderm traits are combined into one module. *Right*: Classes of the $A_{Eq.(1)}$ matrix considered in the analyses of the *Ferula* data. Models 7, and 8 are described in Item 4. The numeric value, 0, implies that the particular entry is constrained to be 0 in the estimation procedure and +, that it is constrained to be positive. Entries denoted by ? are free to vary over the whole real line, they are not constrained to be equal to each other in the matrix. The models are indicated by the numbers to the left of the matrices. In model 8, the matrices A are constrained to be “*DecomposablePositive*.” The rows and columns in the matrices correspond to ratio of canals (row/column 1), periderm thickness (2), wing area (3), wing thickness (4), and fruit mass (5).

We compared eight models with custom setups for the A matrix reflecting various degrees of adaptive parcellation of three primary functions of fruits (Fig. 3). These models may be assigned to the four categories described below. Each model was run with $\Sigma_{yy}$ set as diagonal or as upper triangular, and with or without the measurement error.

1. Functional allometry model (no. 1 in Fig. 3). Here, fruit mass affects the primary optima of both defensive and dispersal traits. This assumption is congruent with the empirical evidence that seed size, which directly influences offspring quality, is usually the main target of selection, whereas the remaining characters, representing other functions, are allometrically related to mass. We also assume that within the functional groups, the traits are correlated, affecting each other’s optima.
2. Parcellation models (nos. 2–4) with various combinations of parcellation of the three primary functions. In model 2, fruit mass (a proxy for seed size) affects only dispersal characters; in model 3, it acts only on defensive traits, whereas in model 4, the three primary functions do not alter each other’s optima.
3. Developmental allometry models (nos. 5 and 6) assume that all anatomical traits that measure *de facto* different characteristics of the fruit wall evolve together affecting each other’s optima, whereas wing area evolves independently from wing thickness. Seed provision affects wing area model in 5 and fruit wall traits in model 6.
4. This category includes two extreme models, the first with all traits evolving independently, i.e., the matrix A was set as diagonal (model 7), and the second with all traits affecting each other’s optima (model 8). With these setups, we wanted to examine if there is a bias toward the simplest model as suggested by simulations (see “Model selection efficiency and quality of parameter estimates”) or toward the most parameter-rich model as suggested by Adams and Collyer (2018).

Because of the small sample size (up to 6 mericarp measurements per species, but 16.7% and 55.1% species had only 1 or 2 replicates respectively) that prevents the reliable estimation of measurement variance for each species (the square of standard error of the species average), we assumed that the within-species variance of each variable was identical for all species (see Ives et al. 2007; Labra et al. 2009; Grabowski et al. 2016). This assumption leads to estimation of the variance for each variable as a sample-size-weighted average of the sample variances of each species, calculated according to Eq. (S.1), by setting $\Sigma_{W_{i}}=s_{wi}^{2}$, where $s_{wi}^{2}$ is the sample variance of species i.

Each model was run 1000 times starting from different starting points obtained from preliminary analyses in which A was set to “*DecomposablePositive*.” The analyses were then sorted by the $AIC_{c}$ values and the run with the smallest value was considered as the best estimate of model parameters. We also performed a simulation study (Appendix SD) to examine the ability to distinguish between models by comparing the two best models under our *Ferula* phylogeny, and then we checked how the result would change as the number of species grows. To do this, we simulated data according to the BM, and two best found models on the *Ferula* phylogeny and then on simulated pure-birth trees with 128, 512, 1024, and 2048 tips. For each model and tree setup we simulated 100 datasets. Then, for each dataset we re-estimated parameters under BM and the two best found models. We considered setups with and without measurement error.

Although in both sets of analyses of empirical data, with and without measurement error, model 7 (matrix A diagonal) was the best, according to $AIC_{c}$, there was a difference in the parameterization of $\Sigma_{yy}$. Without measurement error present, the best model was with $\Sigma_{yy}$ upper triangular (traits correlated through noise), whereas when measurement error was included, the model with $\Sigma_{yy}$ diagonal was favored (all traits are independent). This implies different biological interpretations for the best models (see cases *a* and *b* in “The multivariate Ornstein–Uhlenbeck process”) and shows that contrasting results are possible when different sources of error are not considered in a study. Here, we will examine in greater detail the results from analyses with measurement error as its omission can lead to errors in parameter estimation (Hansen and Bartoszek 2012). However, in addition to model 7, we will also consider model 4 as a plausible one for three reasons. First, it shows up as the second best model with an $AIC_{c}$ value greater than the best model’s one by 9.115. This difference places these models in the “inconclusive region” (see discussion in “Model selection strategies” concerning the work of Burnham et al. 2011). Second, our previously introduced simulation study (“Model selection efficiency and quality of parameter estimates”) indicated a bias toward simpler models with diagonal A. This argument also speaks in favor of model 4. Lastly, there is strong domain-specific knowledge in favor of model 4, it is one of the parcellation models in which all three primary functions of the fruit (i.e., protection, provision, and dispersion) evolve independently from each other.

Our performed simulation showed that some model pairs are distinguishable even with a small number of tip taxa, whereas for others pairs this is difficult, even with large trees. With some model pairs, e.g., 4DmP/4UmP (we use the notation true/competing, see Tabs. S.5 and S.6 for explanation of abbreviations) there is consistent (but slow) improvement with sample size. In other cases, e.g., BMm, distinguishability of the true model seems to decrease with the number of tips. In Table S.7, we can see that numerical errors become more and more evident with the increase in tree size; however with measurement error included they do not take place. Based on the model selection results presented in Table 4, we care most about distinguishing model 4U from 7U and 4Dm from 7Dm (in the 78 tips case). We can see that for the tree and measurement error from the *Ferula* study, model 7 (the simpler one) is easily identifiable, whereas model 4 is not (Table 3). As the sample size increases, the misclassification rates for model 4 decrease (albeit slowly), whereas for model 7 increase.

**Table 3:** Comparison of model identifiability concerning the two pairs of best competing model with measurement error: models 4 and 7 with diagonal $\Sigma_{yy}$. This is a subtable from Table S.6. In the row names, we use the notation true/competing model. The D indicates that $\Sigma_{yy}$ is constrained to be diagonal, P that A’s diagonal is forced to be positive, and *m* that measurement error was included. Each fraction is the result of successful 100 simulation-reestimation repeats. When n=78, the tree is constant across all simulations, the same as the *Ferula* tree; the measurement error is also identical to the one in the *Ferula* study. For greater n, the trees are simulated as pure birth trees and measurement error variance is sampled according to Alg. S.1.

	Number of tips					
Model pair	78	128	256	512	1024	2048
4Dm/7Dm	0.05	0.19	0.37	0.41	0.43	0.5
4Dm P/7Dm P	0.06	0.28	0.28	0.44	0.42	0.53
7Dm/4Dm	0.96	0.78	0.68	0.63	0.57	0.59
7Dm P/4Dm P	0.95	0.75	0.71	0.63	0.54	0.47

**Table 4:** The best $AIC_{c}$ scores along with the log likelihood (Loglik) and $R^{2}$ values for eight models analysed with diagonal and upper triangular $\Sigma_{yy}$ as well as with and without measurement error included. The best two models discussed in the main text were marked in bold.

Without measurement error								
	$\Sigma_{yy}$ diagonal				$\Sigma_{yy}$ upper triangular			
Model	Loglik	$AIC_{c}$	$R^{2}$	dof	Loglik	$AIC_{c}$	$R^{2}$	dof
1	−288.524	626.073	0.0019	23	−210.019	492.360	0.0019	33
2	−277.220	598.957	0.0021	21	−209.727	487.012	0.0019	31
3	−279.100	602.717	0.0023	21	−207.453	482.464	0.0024	31
4	−276.067	592.193	0.0018	19	−205.480	473.808	0.0022	29
5	–255.185	557.134	0.0016	22	–207.125	484.182	0.0023	32
6	–285.483	622.267	0.002	24	–208.809	492.341	0.0021	34
7	–291.323	613.933	0.0016	15	–205.480	**464.542**	0.0022	25
8	–271.311	619.761	0.001	35	–203.637	509.344	0.0022	45
BM	–	–	–	–	–238.132	518.547	0.83	20

With measurement error								
	$\Sigma_{yy}$ diagonal				$\Sigma_{yy}$ upper triangular			
Model	Log-lik	$AIC_{c}$	$R^{2}$	dof	Log-lik	$AIC_{c}$	$R^{2}$	dof
1	–1169.295	2387.614	0.0024	23	–1169.411	2411.144	0.0024	33
2	–1169.379	2383.275	0.0023	21	–1169.190	2405.938	0.0024	31
3	–1169.106	2382.730	0.0024	21	–1169.373	2406.303	0.0024	31
4	–1169.187	**2378.434**	0.0024	19	–1169.363	2401.573	0.0024	29
5	–1169.972	2384.709	0.0024	22	–1169.460	2408.852	0.0023	32
6	–1169.370	2390.038	0.0023	24	–1169.629	2413.982	0.0024	34
7	–1169.016	**2369.319**	0.0024	15	–1169.126	2391.833	0.0024	25
8	–1168.928	2407.855	0.0024	35	–1168.928	2439.925	0.0024	45
BM	—	—	—	—	–1169.527	2381.337	0.95	20

The $AIC_{c}$ values and simulation study results suggest that models 4 and 7 with diagonal $\Sigma_{yy}$ are plausible choices. First, we look into model 7 with diagonal $\Sigma_{yy}$. This model implies independent evolution of all measured traits irrespective of their functional module affiliation. Because it does not describe any particular adaptive hypothesis (no predictors), each trait evolves around a single central state. In this case, the half-life which is interpreted in adaptive scenarios as the time to move half the distance from the ancestral state to the optimum, can be taken as a measure of overall phylogenetic signal (Hansen 2012). The phylogenetic signal in the trait can come from two different sources: phylogenetic inertia or from the distribution of predictor variables on the phylogeny (Labra et al. 2009). Thus, a model with a diagonal A and $\Sigma_{yy}$ may serve as a null model displaying the overall phylogenetic signal for each trait. In model 7, although uncorrelated, the traits responsible for fruit protection, i.e., the proportion of oil ducts in periderm and periderm thickness, evolve the quickest with respective half-lives of 1.32% and 5.08% given here as percentage of tree height in the directions pointed by eigenvectors, which are the same as the traits’ directions as A is diagonal. Assuming that the root height is approximately 4–5 Ma according to molecular dating (Banasiak et al. 2013), the half-life converts to 52–65 ka and 200–250 for each trait respectively. Taking into account that all species of *Ferula* are long-lived perennials, the phylogenetic effect here is weak as it takes no more than several thousands of generations to half the effect of the ancestral state. Dispersal traits, on the other hand, evolve slower with wing area and wing thickness having half-lives of 13.36% and 30.04%  of tree height, respectively. Fruit mass, the only trait representing provision functionality, had a half-life of 15.45% of tree height.

In model 4, traits within functional modules co-evolve adaptively enhancing their functional roles. However, this relationship is not symmetrical. For example, in the dispersal module, the slope of the evolutionary regression between wing area and wing thickness equals −7.498, whereas the slope of the evolutionary regression in the opposite direction is −0.001. However, the half-lives for this module indicated that only 0.09% of tree height is necessary to move half the distance from the ancestral state to the primary optimum in the case of wing area (1^st^ eigenvector of A, in which wing area dominates, see Table 5), whereas over 2000% in the case of wing thickness (5^th^ eigenvector, where wing thickness dominates, see Table 5). Although these estimates should be treated with caution due to numerical problems (see above), the result suggests a much faster adaptation of wing area than wing thickness. In the protection module, we can observe a similar pattern of slope differences—the proportion of oil ducts in periderm had a stronger effect on periderm thickness than vice versa (slope −0.257 vs. slope −0.07). In this case, however, the half-lives are longer (12.09%) and similar based on the two eigenvectors dominated by loadings corresponding to traits from this module (3^rd^ and 4^th^ eigenvectors, see Table 5). Within both protection and dispersal modules, we can observe negative relationships between traits’ optima (negative correlation coefficients), which may indicate trade-offs between characters. The third, provision module, represented here only by fruit mass, showed fast adaptation with 3.7%  of tree height, which converts to 148–185 ka or several thousands of generations. Notice, however, in Table S.20 that in one case the parametric bootstrap confidence intervals do not cover the estimated value of the regression parameter shown here. This can be one of the consequences of considering such a high-dimensional model with so few observations.

**Table 5:** Phylogenetic half-lives with parametric bootstrap (1000 bootstrap replicates) confidence intervals (CI) reported here as percentage of tree height in the eigenvector directions for the best models 7 and 4. The abbreviations relate to measured fruit traits: ratio of oil ducts to length of space between ribs (ROD), mean periderm thickness (MPT), wing area (WA), wing thickness (WTH), and fruit mass (FM) (see also Fig. S.20). Notice that in some cases the bootstrap CIs do not cover the estimated values, illustrating the known difficulties of estimating the parameter as already shown in the one dimensional case (Cressler et al. 2015).

Model 7	Directions (eigenvectors)				
	${\vec{e}}_{1}$	${\vec{e}}_{2}$	${\vec{e}}_{3}$	${\vec{e}}_{4}$	${\vec{e}}_{5}$
ROD	0	1	0	0	0
MPT	1	0	0	0	0
WA	0	0	1	0	0
WTH	0	0	0	0	1
FM	0	0	0	1	0
Half-life	1.32%	5.08%	13.36%	15.45%	30.04%
(CI)	(0.10%, 26.26%)	(3.52%, 40.06%)	(17.67%, 60.12%)	(23.61%, 66.22%)	(25.19%, 68.82%)

Model 4	Directions (eigenvectors)				
	${\vec{e}}_{1}$	${\vec{e}}_{2}$	${\vec{e}}_{3}$	${\vec{e}}_{4}$	${\vec{e}}_{5}$
ROD	0.00	0.00	–0.33 – 0.54*i*	–0.33 + 0.54*i*	0.00
MPT	0.00	0.00	0.78	0.78	0.00
WA	0.99	0.00	0.00	0.00	0.00
WTH	–0.01	0.00	0.00	0.00	0.99
FM	0.00	1.00	0.00	0.00	0.00
Half-life	0.09%	3.7%	12.09%	12.09%	2207.8%
(CI)	(0.03%, 25.91%)	(2.21%, 48.03%)	(7.92%, 58.94%)	(19.42%, 66.35%)	(10.87%, 128.14%)

An interesting result from the analyses is also an observed high variation in the values of likelihood function between different runs of the same model. With measurement error, the best model 7 with diagonal $\Sigma_{yy}$, had the log-likelihood varying from −1463 to −1169.016. Even bigger differences among runs were obtained for models without measurement error included. For example, for model 7 with diagonal $\Sigma_{yy}$, the log-likelihood varied from −1558198.186 to −291.323. This serves as an important reminder that many repetitions of the same model using different random seeds and starting points are necessary to obtain the best possible estimates of model parameters.

In the Section “Model selection efficiency and quality of parameter estimates”, we noticed that there is a bias towards simpler OU models in analyses. A diagonal A model (model 7; Fig. 3) was found to be best by $AIC_{c}$, and in simulation results presented in Table 3 a similar preference for simpler models, with small sample sizes, is visible. However, as the number of tips increases, model 4 becomes more identifiable, albeit very slowly. On the other hand, with the increase of tips, model 7 becomes less identifiable. On A’s diagonal for both models 4 and 7, we have large values (the trees are always scaled to height 1), implying short half-lives/rapid loss of ancestral signal/fast adaptation to the optimum. Hence, as in both cases the tip sample will be oscillating around the global optimum, additional parameters (with a large sample) can lead to overfitting, for which $AIC_{c}$ does not penalize sufficiently for.

If one looks in detail at Table S.6, one can see that measurement error can have a profound effect on being able to distinguish between models. When simulations were done without it, the BM model is always correctly identifiable. Models 4D and 7D are still hard to distinguish between each other. It is usually possible to distinguish between diagonal and upper triangular $\Sigma_{yy}$ matrices, especially with the *Ferula* tree. It seems also possible to distinguish between models 4 and 7 assuming an upper–triangular $\Sigma_{yy}$ matrix. However, what can also be seen is that as the number of tips increases, there is a tendency towards the diagonal A model 7, which is in line with our observations from the simulation study in “Model selection efficiency and quality of parameter estimates”. We underline that it seems that the problem of identifying the correct structure of A from comparative data is a subtle one and might require the development of new decision methods. On the other hand, we do not notice issues with rejecting or correctly accepting models where BM traits are present.

Without measurement error, as the sample grows, especially for the Brownian motion model, numerical errors seem to be more and more common. In fact, with 2048 tips so many as 881 trees and trait simulations had to be performed in order to obtain a sample of size 100. A possible explanation for these numerical errors are too short tip branches which cause singularities. When measurement error is present, such numerical problems are neither observed for BM nor for OU models. This supports that the problem is mainly with short tip branches. Measurement error additively increases the variance of change along a tip branch. If a tip branch is so short that for a given parameter the covariance matrix of change along a tip branch is singular, then adding the variance of measurement error removes this singularity. However, this comes at a price. In Fig. S.10, we can see that without measurement error, the estimator of $\Sigma_{xx}$, from the accepted simulation samples, seems to behave consistently.

On the other hand, when measurement error is present, parameter estimation errors are larger and improvement (other than the decrease of estimation variance) cannot be observed. Similarly, for the OUOU models, parameter estimation error is much smaller without measurement error. With measurement error, the misclassification rate of BM increased. Given a single optimum and ultrametric tree, the collection of measurements for tip species is identically (but not independently) distributed. If the noise from measurement error dominates and masks signal, especially dependencies between observations, then information criteria could favor the simplest model that can fit a common mean and variance. As misclassification rates with measurement error present seem to all exhibit similar behavior, it could be that a given level of measurement error will induce convergence to a particular limit of the misclassification rate.

Many plants develop seed packaging structures, which play various roles throughout the seed life span. Hard encapsulation, as in the case of *Ferula*, protects seeds and assists in dispersal with the help of specialized structures such as wings. Generally, our analyses supported two different parcellation models indicating independent evolution of all or some of studied fruit traits. The possible scenario for evolution of these quasi-independent functional modules in fruits can be inferred from empirical studies showing that selection pressure from various agents often conflicts. An interesting example comes from research on tree squirrels (*Tamiasciurus*), which are important seed-eaters influencing conifer reproductive strategies (Benkman 1995). The study showed that limber pine (*Pinus flexilis*) coming from regions affected and unaffected by tree squirrels did not differ in seed kernel mass but did in seed-coat characters. This shows that the protective and provision functions evolved independently. The former was the main target of directional selection although the latter was under stabilizing selection as abiotic factors influencing germination—that are important selective agents of kernel mass—do not differ between these regions (Benkman 1995). Although this study showed the conflicting selection on seed functions, the evolution was rapid and likely free of any constraints as squirrels were extirpated in the studied region approximately 12,000 years ago. In the case of interspecific comparative analysis of fruits of *Ferula*, the rate of co-adaptive changes in fruit traits is also fast—taking no more than several thousand of generations. This is in agreement with observations of other species from the subfamily Apiaceae, that show, although indirectly, rapid change in quantitative traits of fruits. For example, in the genus *Chaerophyllum* belonging to the same tribe as *Ferula*, there is no quantitative character of fruit that characterizes clades assigned to taxonomic sections (Piwczyński et al. 2015). The principal component analysis (PCA) showed, that in many cases, sister taxa have more divergent traits than distantly related ones leading to complete overlap between convex hulls delimiting sections. Similar results were obtained by Wojewódzka et al. (2019). The Authors searched for common combinations of anatomical and morphological traits that are associated with switches among different dispersal syndromes. The results show no such combinations with overlapping convex hulls between species representing various dispersal strategies and various phylogenetic affinities. This may point to mixed dispersal strategies as suggested by the Authors, but it is also in agreement with quasi-independent evolution of traits associated with various functions in the fruit as suggested by analysis of *Ferula* data. It is reasonable, for example, to assume that wind dispersal species may be under a wide range of selection pressures, often in opposite directions, on protective traits of fruit. Modules may help in optimizing each trait independently by selection (Hansen 2003).

Another set of interesting results from model 4 is the negative relationship between traits within functional modules. In the case of dispersal, this relationship is expected from aerodynamic theory which states that any diaspore size, having any settling velocity, can be dispersed to a given distance if the shape of falling object is sufficiently modified (Niklas 1994). In our example, it can be argued that the increase in wing area is counteracted by a decrease in wing thickness to optimize area–mass relationship. Unfortunately, the estimation of regression coefficients was imprecise—precluding deeper analysis. The second negative relationship occurred in the protection module that may be explained by trade-off between costly traits. Generally in Apiaceae, various forms of endosperm protection do not occur together. For example, species having thickened cuticule possess reduced canals and bundles (Spalik et al. 2001). In line with this observation, the increased protection by thickening of periderm leads to reduction of canals without harmful consequences.

As pointed out by Niklas (1994), evolution of wind dispersed fruits seems to be counterintuitive. Plants add extra mass to seeds potentially reducing dispersal capacity. However, this excess of tissues may have higher evolutionary potential, expanding the range of phenotypic characters available for selection, in comparison with a seed coat. In addition, fruit walls can offer an extra protective barrier against seed predators. This increase in evolutionary potential may be realized by modularity, which may sufficiently reduce the consequences of conferring extra mass to seeds.

Our analyses provided several important observations for **mvSLOUCH** users. First, the user must pay attention to the quality of the data. In a typical comparative analysis, the sample size differs from species to species even by orders of magnitude. This, as shown in our analyses, can lead to imprecise estimation of measurement error and, in consequence, problems with estimation of model parameters (Table 4). From our simulations, we can also observe that measurement error can have a big effect on estimation. Our analyses aimed to illustrate **mvSLOUCH**’s possibilities, but to make firm conclusions about the genus *Ferula*, we can see that 78 species are too few, and measurement error is too high (too few replicates per species). This is most evidently seen in the (marginal, 2.5% and 97.5% quantiles) parametric bootstrap confidence intervals presented in Tables. S.19 and S.20 for the correlation and regression coefficients. In all the non-zero (by model definition) cases, the confidence intervals cover zero. Our simulations show that with the given sample the more complex model 4 is not identifiable. However, as we increase the tree size, non-ignorable numerical issues start to appear. The third observation concerns the interpretation of model parameters, which is impossible without a clear hypothesis. The software cannot be used for a “phylogenetic correction,” the species’ joint evolutionary history should be used to test specific hypotheses motivated by our theories and knowledge of the considered system. Estimated parameters should therefore be interpreted with a link to what drove the particular study setup.

## Discussion

Our simulation studies, Appendix SC and Appendix SD, found that if there is any bias in model identifiability, it will be in the direction of simpler models (opposite to the conclusions of Adams and Collyer (2018), based on a BM simulation and BM, OU reestimation study). On the other hand, our simulation from the *Ferula* study showed that as the sample size increases, information criteria can prefer more complex OUOU models over simpler OUOU ones (Table 3, rows where data was simulated under model 7). However, as discussed already, the chosen parameters are difficult ones for the estimation procedures. For small n, the BM could outperform more complex OUOU, OUBM models. As n increased, this preference toward BM disappeared for all models. OUBM models are distinguishable from OUOU models, but distinguishing between different types of A matrices inside the OUBM class might not be that straightforward. However, again the main error seemed to be in the case of preferring simpler models—diagonal A when simulated under a non-diagonal one. The most striking issue is that the diagonal A–diagonal $\Sigma_{yy}$ (OUOUs1) model is consistently preferred over the non-diagonal A–diagonal $\Sigma_{yy}$ (OUOUs2) model. It is known from previous, one-dimensional, studies that even the scalar counterpart of A is difficult to estimate (Cressler et al. 2015). Our multivariate simulation–reestimation study confirms this, with non-diagonal A there were many outliers with high relative errors for this parameter (and others). Therefore, in the multivariate case, it seems to be an open question if there is a parameter-identifiability issue for A, or more data (i.e., larger n) is required to justify the choice of the model with more parameters. Hence, a practitioner should always be careful when the OUOU model with diagonal A is indicated and should weight the understanding of the system under study. In all model cases, some parameters are easier to estimate—those corresponding to the diffusion parameter, $\Sigma_{xx}$, $\Sigma_{yy}$, Σ. For these, the boxplots in Appendix SC suggest consistency. The drift parameters, entries of A; A’s associated eigenvalues, eigenvectors, half-lives, as already discussed, are more difficult to estimate, especially if it is non-diagonal. Covariance, correlation, and regression parameters also tend to have much variability in their estimation. Finally, it has to be stressed that the simulations did not indicate problems with identifying the main class of models. From n=128, major problems with discriminating BM from OUOU from OUBM are not visible. Perhaps it is difficult to identify the type (based on the A matrix’s type) of model in some situations, but the main dynamics seemed identifiable.

It is also worth pointing out that Mitov et al.’s (2020) posterior-quantile validation did not find any issues with **PCMBase**’s loglikelihood values. Mitov et al.’s (2019) very extensive simulation study also does not indicate any serious issues with estimation nor model identifiability of the **PCMFit** package, which uses the same likelihood computation engine provided by **PCMBase** and **PCMBaseCpp**.

To compare our simulations study with Adams and Collyer’s (2018), when simulating under the BM model, we restrict our simulations to independent traits. Re-estimation was done under multiple setups but two are worth discussing. One (OUOU5 in Table S.2) was with A assumed as general invertible and $\Sigma_{yy}$ upper triangular. This is the default, but highly unrecommended, **mvSLOUCH** setting that Adams and Collyer (2018) used. It has the property that it does include the least possible assumptions and hence a priori biological hypotheses about the relationships between traits. Having it as a default will not bias the user towards any particular hypothesis and hopefully encourage to explore different ones. However, it is extremely unstable to estimate under and, from our experience, tends to get very easily stuck in local optima and is therefore not recommended. We also take models with A eigendecomposable with positive real eigenvalues and diagonal positive or free, and upper-triangular $\Sigma_{yy}$ (OUOU4, OUOU4P, OUBM4, OUBM4P in Table S.2). The latter setups, we believe, are fairer towards **mvSLOUCH** than Adams and Collyer’s (2018) use of the default settings. The numerics seem, from our experience, to be significantly more stable than in the default case and, we re-iterate, PCM studies should be carried out with some biological hypotheses in mind, corresponding to particular parameter settings.

We ran estimation both with A’s diagonal constrained to be positive or free to vary. When the diagonal was assumed positive, then the diagonal values were exponentiated after the matrix was calculated from its eigendecomposition parametrization. As positive real eigenvalues do not imply a positive diagonal, we wanted to see what implication a positive diagonal constraint could have on the estimation. On the one hand, it will guarantee a correct sign of the diagonal, but on the other, interfere with R’s numerical optimizer’s, *optim()*, search path. And in fact this was observed. Under the OUBM model, for non-diagonal A, forcing the diagonal to be positive reduced model selection abilities. In the OUOU case, such an issue was not observed.

The simulation study based on parameters estimated from the *Ferula* data brought up further issues that need to be heeded when using PCMs. Measurement error can result in decreasing quality of parameter estimates and make deciding between models difficult, but must still always be included in comparative analyses. When trees become large, numerical problems can become more of an issue. However, the observed numerical problem is due to very short tip branches, hence it might not be present at all in real estimated phylogenies. Short tip branches arise naturally when trees are simulated under birth-death models conditioned on a particular (large) number of tips. Hence, it becomes a question, whether, despite their simplicity and mathematical elegance, these are the optimal ways of generating trees for simulation studies of PCMs. Even if the Brownian motion model is not of interest, the user of **mvSLOUCH** is advised to consider it. Closed form estimation formulæ will pick up the numerical issues due to short branches leading to tips (by returning infinite information criteria) and indicate that the estimation results under the OUOU models require careful scrutiny. A possible way of dealing with such a situation is to drop species with short pendant branches (if there are few). Alternatively, if there would be many such situations, multiple random subsampling with, e.g., subsequent model averaging can be employed. However, the choice of action is particular to the situation, and is up to the user. Based on our simulations here, we recommend that an applied PCM user should not only look at the single best (according to their chosen measure) model but also explore those in the “plausible set,” taking into account domain knowledge and estimated parameters (models could have nearly the same parameter values e.g., non-zero values are very close to zero). A simulation study limited to the plausible set of models can indicate whether there is distinguishability between them, and a parametric bootstrap will show parameter identifiability. With the analyses and simulation studies here, we have only touched the tip of the iceberg and as algorithmic improvements in PCM software have reduced running times and numerical problems (or made them clearer), we will be able to design more and more complex simulation studies that will give better understanding of estimation possibilities. Hopefully, it will be possible to approach this from the mathematical direction to have analytical results on the inference methods.

## Supplementary Material

The GitHub repository https://github.com/krzbar/KJVJMRKK_mvSLOUCH contains R scripts, data, random seeds and simulation outputs used in this work. These files are also available from the Dryad Digital Repository: http://dx.doi.org/10.5061/dryad.sj3tx9656.
